# One Year Real-World Use of the Control-IQ Advanced Hybrid Closed-Loop Technology

**DOI:** 10.1089/dia.2021.0097

**Published:** 2021-09-01

**Authors:** Marc D. Breton, Boris P. Kovatchev

**Affiliations:** Center for Diabetes Technology, University of Virginia, Charlottesville, Virginia, USA.

**Keywords:** Continuous glucose monitoring, Automated insulin dosing, Closed-loop control, Time in range

## Abstract

***Background:*** The t:slim X2™ insulin pump with Control-IQ^®^ technology from Tandem Diabetes Care is an advanced hybrid closed-loop system that was first commercialized in the United States in January 2020. Longitudinal glycemic outcomes associated with real-world use of this system have yet to be reported.

***Methods:*** A retrospective analysis of Control-IQ technology users who uploaded data to Tandem's t:connect^®^ web application as of February 11, 2021 was performed. Users age ≥6 years, with >2 weeks of continuous glucose monitoring (CGM) data pre- and >12 months post-Control-IQ technology initiation were included in the analysis.

***Results:*** In total 9451 users met the inclusion criteria, 83% had type 1 diabetes, and the rest had type 2 or other forms of diabetes. The mean age was 42.6 ± 20.8 years, and 52% were female. Median percent time in automation was 94.2% [interquartile range, IQR: 90.1%–96.4%] for the entire 12-month duration of observation, with no significant changes over time. Of these users, 9010 (96.8%) had ≥75% of their CGM data available, that is, sufficient data for reliable computation of CGM-based glycemic outcomes. At baseline, median percent time in range (70–180 mg/dL) was 63.6 (IQR: 49.9%–75.6%) and increased to 73.6% (IQR: 64.4%–81.8%) for the 12 months of Control-IQ technology use with no significant changes over time. Median percent time <70 mg/dL remained consistent at ∼1% (IQR: 0.5%–1.9%).

***Conclusion:*** In this real-world use analysis, Control-IQ technology retained, and to some extent exceeded, the results obtained in randomized controlled trials, showing glycemic improvements in a broad age range of people with different types of diabetes.

## Introduction

Diabetes mellitus is one of the best quantified human conditions: elaborate models describe the action of the metabolic system, real-time signals, such as continuous glucose monitoring (CGM), are readily available, insulin delivery can be automated, and advancing artificial pancreas (AP) technology is increasingly capable of controlling blood glucose fluctuations in a patient's natural environment. Consequently, in the past 30 years, the treatment of diabetes progressed from occasional clinical encounters and review of hemoglobin A1C (HbA1c) once in several months, through daily insulin adjustments using episodic self-monitoring, to fine-tuning of treatment decisions driven by CGM and automated closed-loop control (CLC), a.k.a. the AP.^1^

In 2004–2007, the first attempts to automate glucose control using CGM and Continuous Subcutaneous Insulin Infusion (CSII) were made with Proportional Integral Derivative (PID) and Model Predictive Control (MPC) algorithms.^2–4^ Over the subsequent decade, a variety of algorithms were evaluated, with the bulk falling into either PID, MPC, or Fuzzy Logic paradigms^5^: Between 2016 and 2020, PubMed included >120 publications per year related to engineering and clinical testing of various AP systems. Systematic reviews and meta-analyses are now available^1,5,6–9^ and an international consensus was reached on the use of glycemic control metrics for the interpretation of CGM and AP data.^10,11^

In 2016, a letter to *JAMA* announced the first pivotal trial of a new hybrid closed-loop system—the MiniMed 670G (Medtronic, Dublin, Ireland).^12^ This first study did not have a control group, focusing solely on the safety of the system, which was subsequently approved by the Food and Drug Administration (FDA) for clinical use. In the years that followed, a number of long-term randomized clinical trials were reported, assessing AP safety and efficacy for 3–10 months.^13–17^

In 2018–2020, the subject of this article—the new advanced hybrid closed-loop system t:slim X2™ insulin pump with Control-IQ^®^ technology from Tandem Diabetes Care, based on a control algorithm developed at the University of Virginia, was tested in two randomized controlled trials (RCTs) comparing Control-IQ with sensor-augmented pump (SAP) therapy. Both studies were part of the International Diabetes Closed-Loop (iDCL) Trial sponsored by the National Institute of Diabetes and Digestive and Kidney Diseases (NIDDK): NCT03563313 randomized *n* = 168 participants ages 14 years and older to Control-IQ versus SAP, all of whom completed the 6-month trial. The study met all of its predefined primary and secondary outcomes, resulting in 11% increase in the time within 70–180 mg/dL, reduction in the time <70 mg/dL by 0.9% without any severe hypoglycemic episodes, and reduction of HbA1c by 0.3% on Control-IQ compared with SAP.^18^ NCT03844789 randomized *n* = 101 participants ages 6–13 years old and achieved outcomes similar to those in adults: Control-IQ compared with SAP resulted in 11% increase in the time within 70–180 mg/dL, 0.4% reduction in the time <70 mg/dL without severe hypoglycemic episodes, and reduction of HbA1c by 0.4%.^18^ These trials led to the FDA clearance of this system for clinical use children and adults, ages 6 and up.

In this article, we report data from 1-year of real-world use of t:slim X2 with Control-IQ technology in >9000 users with diabetes, who wore the system for 12 months as part of their regular clinical treatment.

## Research Design and Methods

The Tandem t:slim X2 with Control-IQ technology was commercially released in the United States in January 2020. This advanced hybrid closed-loop system comprises a t:slim X2 insulin pump with an embedded model-based CLC algorithm, an integrated Dexcom G6 CGM, and an infusion set.

### Algorithm action

Control-IQ technology uses CGM and delivered insulin data to predict glucose levels 30 minutes ahead and adjust insulin delivery accordingly, including automatic correction boluses. If sensor glucose values are predicted to drop <112.5 mg/dL, basal insulin is reduced, and when predicted to be <70 mg/dL, basal insulin delivery is stopped. If sensor glucose values are predicted to be >160 mg/dL, basal insulin is increased. If sensor glucose values are predicted to be >180 mg/dL, Control-IQ calculates an automatic correction bolus with a target of 110 mg/dL and delivers 60% of that value. Automatic corrections are allowed up to once per hour as needed. Different treatment targets apply when a Sleep or Exercise Activity is enabled, with for example a gradual tightening of glycemic control during Sleep to 120 mg/dL. If the CGM connection has been lost or stopped for 20 min or longer, Control-IQ will stop automatically adjusting insulin delivery and the pump returns to the active Personal Profile settings until the connection is restored. Once restored, Control-IQ will resume automatically.

### Indications

Control-IQ technology is a prescription device embedded in the software of the t:slim X2 insulin pump and indicated for people ages 6 years and up. Individuals new to Tandem pumps must train with a Certified Diabetes Education Specialist to onboard to Control-IQ technology. For people already using an in-warranty Tandem t:slim X2 pump, starting Control-IQ technology begins with a remote software update, after completion of online educational training. As of publication >100,000 people with diabetes have on-boarded to this system.

### Study population

We performed a retrospective analysis of users of Control-IQ technology in the United States who had descriptive data available in Tandem's Customer Relations Management database and had uploaded their glycemic data—either through Tandem's t:connect uploader or through the mobile app—to Tandem's t:connect web application as of February 11, 2021. The data extracted for analysis were de-identified. Participants consented to the use of their data for research purposes as part of their onboarding to Tandem when initiating their t:connect account. No IRB approval was sought for this retrospective analysis. Data from users who were ages 6 years and above, had at least 12 consecutive months of data available on Control-IQ, and had at least 2 weeks of CGM data with ≥75% CGM availability before Control-IQ initiation were included in the analysis. Any Control-IQ user with data uploaded in t:connect that did not meet these criteria were excluded.

### Outcome metrics

Percent time in closed-loop automation was calculated as the percentage of the total basal rates delivered by the pump, in 5-min increments, which were decided by the Control-IQ algorithm. Descriptive data were organized according to diabetes type, age, gender, time since diabetes diagnosis, glucose management indicator, previous Tandem pump software versions, and time in Control-IQ technology automation. Glycemic outcomes were calculated for all participants who had at least 2 weeks of CGM data with ≥75% CGM availability before Control-IQ initiation and 1 year of CGM data with ≥75% CGM availability after Control-IQ initiation.

The outcomes were calculated by participant, per time period of interest (2 weeks, 1 month, 3 months, 6 months, and 1 year), and the median and interquartile range (IQR) across subjects are reported. CGM values outside the valid ranges of 40–400 mg/dL were filtered out, with glucose value <40 mg/dL and >400 mg/dL being saturated at 40 mg/dL and 400 mg/dL, respectively. No CGM interpolation was performed. Glycemic outcomes are reported regardless of closed-loop status. Outcomes were analyzed using Wilcoxon signed rank test and are reported as median (quartiles). The study was not statistically powered and tests are not corrected for multiple comparisons.

Distribution are presented as either boxplots (a graphical representation including median, mean, quartiles, and extremes for a given outcome) or violin plots, the combination of boxplots with the addition of a rotated kernel density estimation step,^19^ the kernel estimation being saturated at 0% and 100%.

## Results

Overall, 9451 users met the inclusion criteria, of which 83% had type 1 diabetes (T1DM) and the rest had type 2 (T2DM) or other forms of diabetes; and 52% were female (Table 1). Mean age for the sample was 41.9 ± 20.8 years, almost 99% were using Basal-IQ technology (a predictive low glucose suspend system embedded in the t:slim X2 pump) at baseline. After filtering for availability of CGM (≥75% CGM use for the 2 weeks preceding and for the 12 months after Control-IQ technology initiation) 9010 users remained, or ∼95% of the original set.

**Table 1.** Baseline Attributes of System Users with At Least 12 Months of Consecutive Control-IQ Technology Software Data Available

N	9451
Age	41.9 ± 20.8, minimum 6 years; maximum 91 years
Gender	Female: 52% (*n* = 4905)
	Male: 48% (*n* = 4540)
eA1c	7.3%
Diabetes type	Type 1 diabetes: 83% (*n* = 7813)
	Type 2 diabetes: 4% (*n* = 378)
	Not self-reported: 13% (*n* = 1260)
Diabetes duration	Type 1 diabetes: 21.84 ± 20.6
	Type 2 diabetes: 20.76 ± 10.3
Prior insulin pump software	Tandem t:slim X2 pump with Dexcom G5 Mobile CGM: 1.26% (*n* = 119)
	Tandem t:slim X2 pump with Basal-IQ technology: 98.74% (*n* = 9332)

Data presented as mean ± SD or % (*n*)

CGM, continuous glucose monitoring; eA1c, estimated hemoglobin A1C.

**FIG. 1.**
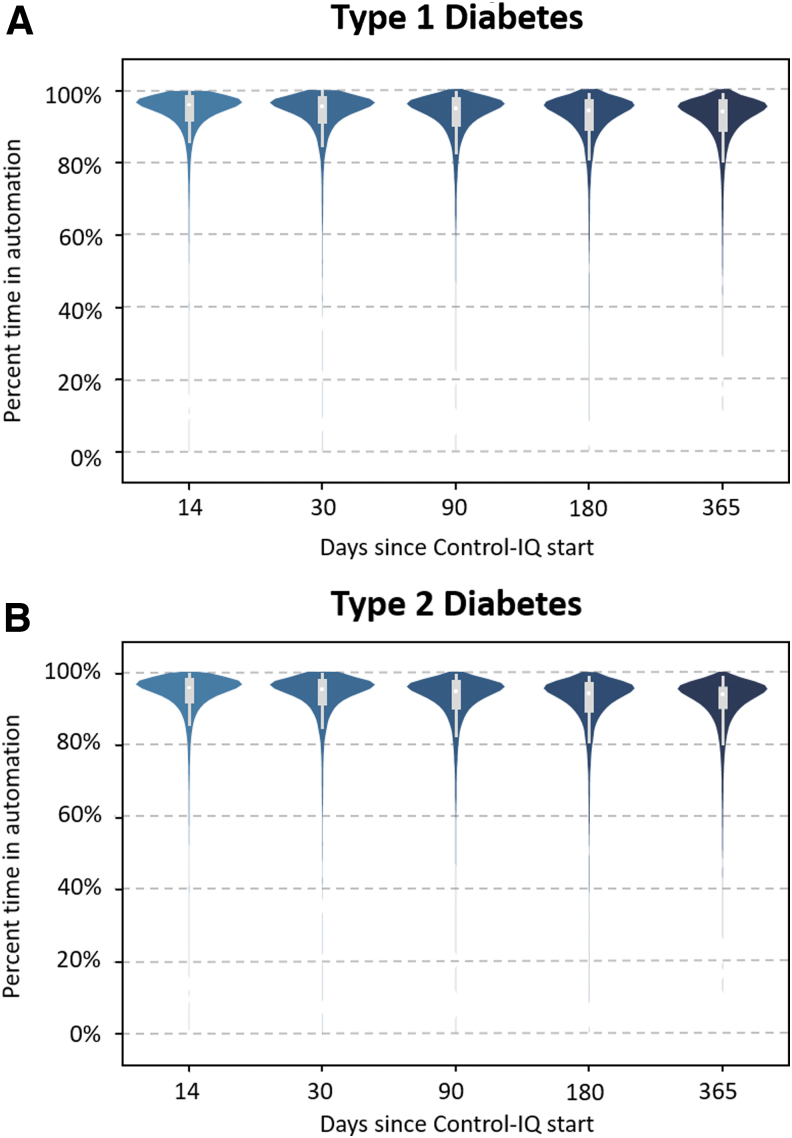
Violin graph representing percent time in automation over 365 days of Control-IQ (CIQ) technology use by type of diabetes (A: T1DM, B: T2DM).

Median percent time in automation was 94.2% [IQR: 90.1%–96.4%] for the entire 12-month duration with no significant changes over time (first 3 months: 95.1%, first 6 months: 94.5%). High automation was observed for both types of diabetes (Fig. 1), and in all age groups with three quarters of each subpopulation above ∼90% automation (Supplementary Fig. S1 and Supplementary Table S1), although adolescents (14–18 years) showed a slightly lower time in automation than the rest of the population (92.5% [88.3–95.0]).

**FIG. 2.**
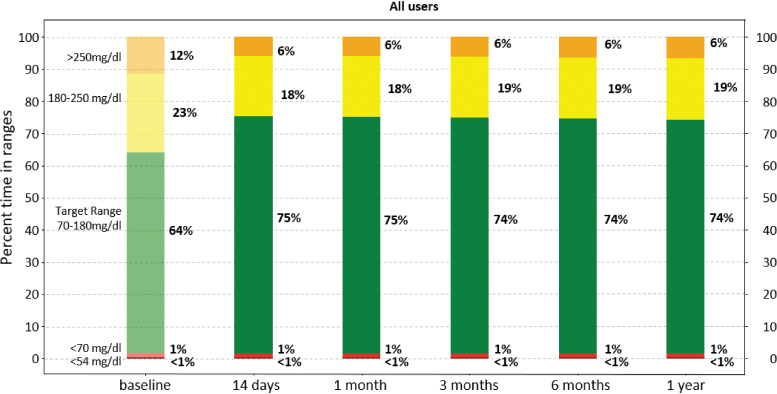
Consensus CGM outcomes for baseline and Control-IQ use over time. CGM, continuous glucose monitoring.

**FIG. 3.**
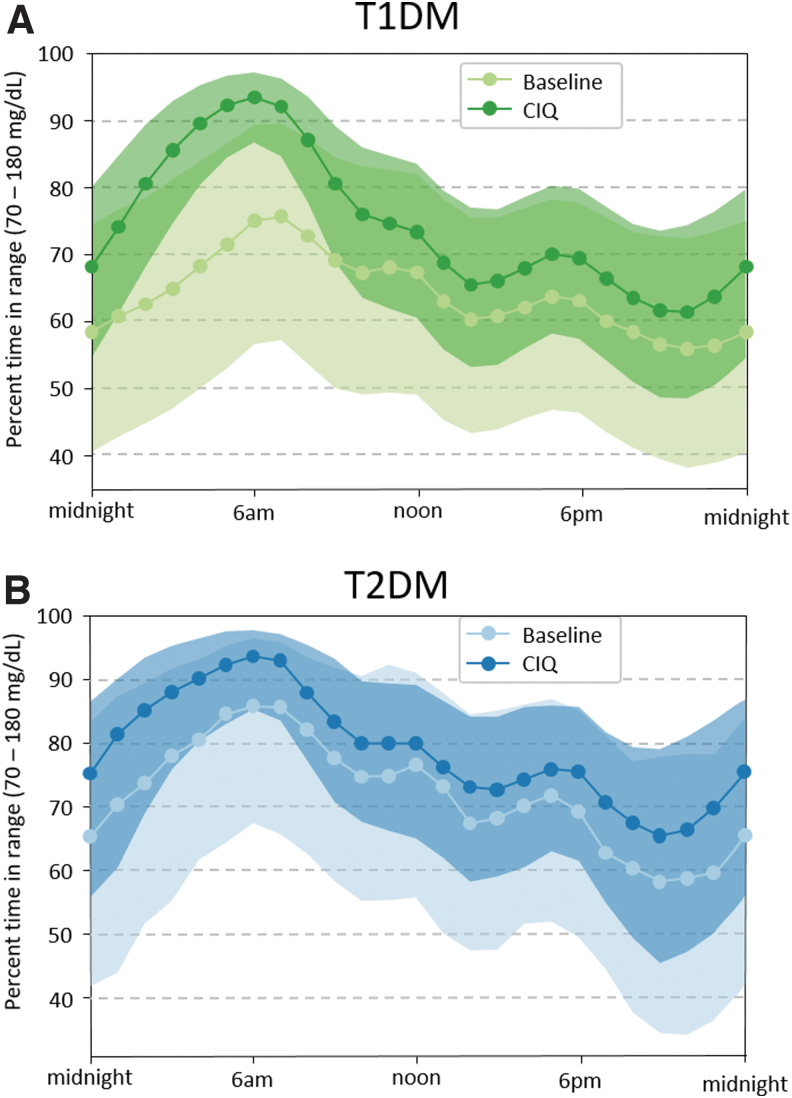
Median (line and markers) and quartiles (envelop) of Time In Range (TIR, 70–180 mg/dL) over time of day by types of Diabetes (A: T1DM, B: T2DM) for Baseline and 1 year of CIQ use.

**FIG. 4.**
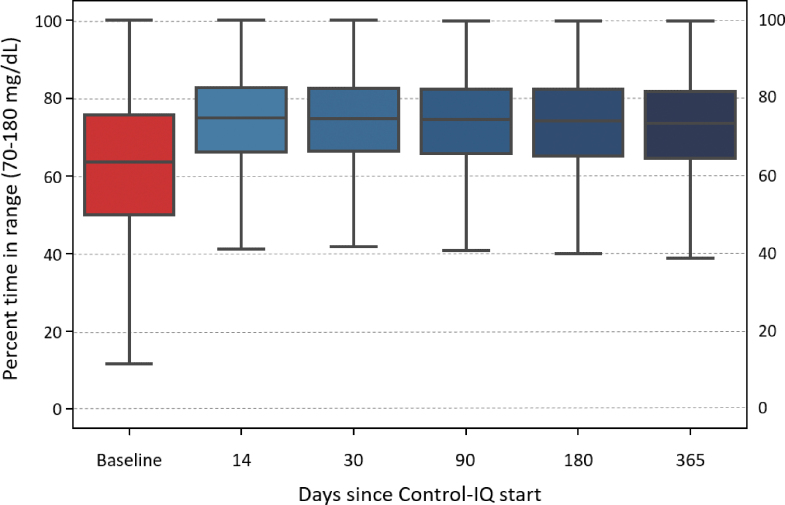
Overall distribution of Time In Range (TIR, 70-180mg/dL) at baseline and over time of CIQ use. Horizontal line shows median and filled rectangle represent the 50% of data within the first and third quartiles, whiskers represent minimum and maximum values.

Glycemic control improved rapidly (within 2 weeks) after initiation of Control-IQ and was maintained for the entirety of the study period (Fig. 2). Specifically, median percent time in range (TIR) (70–180 mg/dL) was 63.6 (IQR: 49.9%–75.6%) at baseline and increased to 73.6% (IQR: 64.4%–81.8%) for 12 months of Control-IQ use (*P* < 0.001) with no significant degradation in time (Fig. 3). Median percent time <70 mg/dL remained consistent at ∼1% (IQR: 0.5%–1.9%, Fig. 4), whereas median percent time <54 mg/dL statistically increased from 0.1% [0.0–0.3] to 0.15% [0.06–0.3]. Mean glucose decreased from 167.5 ± 30.7 mg/dL at baseline to 154.3 ± 20.7 mg/dL for the 12 months after Control-IQ initiation. These improvements were seen for both people with T1DM and T2DM, with median TIR improvements of +10.3% and +8.1%, respectively. Table 2 summarizes the main glycemic outcomes.

**Table 2.** Comparison of Glycemic Outcomes for Users Who Had ≥75% Continuous Glucose Monitoring Use (Baseline vs. 12-Month Use of Control-IQ)

	Baseline (Basal-IQ)	12-mth control-IQ use	P
All users			
No. of participants	9010	9010	
Mean sensor glucose [mg/dL]	164 (146–185)	152 (140–166)	<0.001
Sensor time <54 mg/dL [%]	0.10 (0.00–0.30)	0.15 (0.06–0.30)	<0.001
Sensor time 54–70 mg/dL [%]	0.8 (0.3–1.8)	0.9 (0.4–1.6)	0.053
Sensor TIR [%]	63.6 (50.0–75.7)	73.6 (64.5–81.8)	<0.001
Sensor time 180–250 mg/dL [%]	25.1 (18.0–31.1)	19.7 (14.2–24.3)	<0.001
Sensor time >250 mg/dL [%]	8.1 (2.9–16.7)	4.6 (1.9–9.5)	<0.001
Coefficient of variation [%]	33.7 (30.0–37.6)	32.9 (29.5–36.3)	<0.001
GMI	7.2 (6.8–7.7)	6.9 (5.6–7.3)	<0.001
T1DM users			
No. of participants	7813	7813	
Mean sensor glucose [mg/dL]	163 (141–190)	151 (134–170)	<0.001
Sensor time <54 mg/dL [%]	0.01 (0.00–0.35)	0.02 (0.00–0.4)	<0.001
Sensor time 54–70 mg/dL [%]	0.9 (0.3–1.9)	0.9 (0.5–1.7)	0.123
Sensor TIR [%]	63.2 (49.8–75.1)	73.5 (64.4–81.6)	<0.001
Sensor time 180–250 mg/dL [%]	25.2 (18.2–31.0)	19.7 (14.3–24.2)	<0.001
Sensor time >250 mg/dL [%]	8.3 (3.1–16.9)	4.7 (2.0–9.6)	<0.001
T2DM users			
No. of participants	378	378	
Mean sensor glucose [mg/dL]	158 (138–184)	150 (136–169)	<0.001
Sensor time <54 mg/dL [%]	0.00 (0.0–0.07)	0.04 (0.01–0.10)	<0.001
Sensor time 54–70 mg/dL [%]	0.2 (0.0–0.6)	0.2 (0.0–0.6)	0.337
Sensor TIR [%]	69.9% (55.1–82.6)	78.0% (66.2–86.1)	<0.001
Sensor time 180–250 mg/dL [%]	23.9 (14.6–32.0)	19.0 (12.4–25.5)	<0.001
Sensor time >250 mg/dL [%]	3.6 (0.7–10.4)	2.3 (0.8–6.7)	<0.001

Data are expressed as median (IQR) unless otherwise specified.

GMI, glucose management indicator; IQR, interquartile range; T1DM, type 1 diabetes; T2DM, type 2 diabetes; TIR, time in range.

**FIG. 5.**
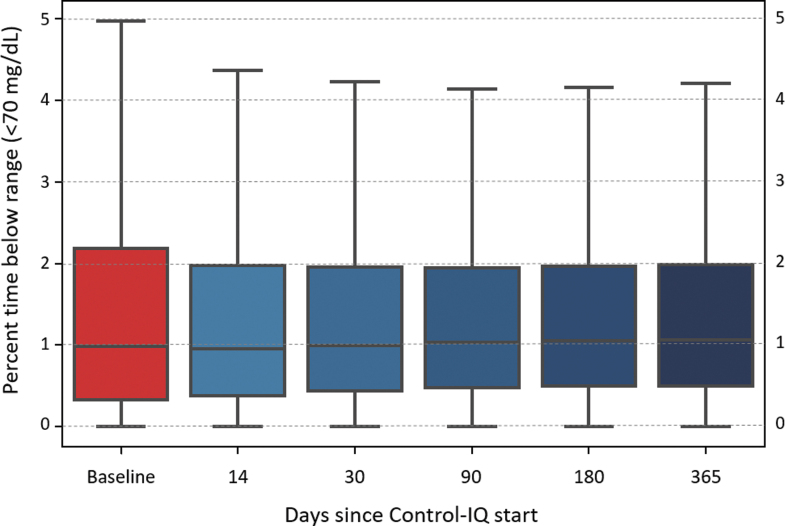
Overall distribution of Time Below Range (TBR, <70mg/dL) at baseline and over time of CIQ use. Horizontal line shows median and filled rectangle represent the 50% of data within the first and third quartiles, whiskers represent minimum and maximum values.

Most of these improvements for T1DM users were driven by profound TIR increase at night, reaching a median >90% between 4 and 7 am, which is a reflection of the control algorithm design; in T2DM users the improvements over baseline were similar throughout the day, with a peak >90% by 4 am (Fig. 5).

**FIG. 6.**
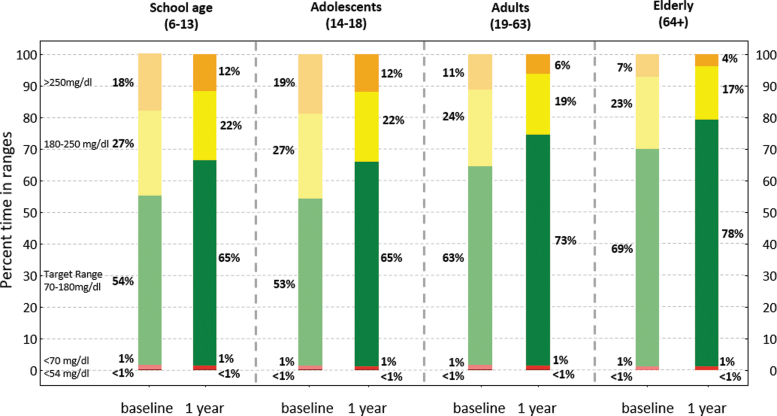
Consensus CGM outcomes for baseline and 12-months Control-IQ use broken down by age groups.

These outcomes were seen across age groups (from +9 in elderly users to +13 for adolescents) (Fig. 6). We observed larger improvements in populations with lower baseline TIR and interpatient variability was clearly reduced (Supplementary Figs. S2 and S3).

## Discussion

This uniquely large data set of AP real-world use confirmed the glycemic control improvement noted during the system pivotal trials^18,20^: in a sample of >9000 patients with diabetes, TIR improved by ∼10 percentage points in people with T1DM (62% vs. 72%, or ∼2.5 h/day improvement) from baseline compared with 11 percentage points for both RCTs. Both RCTs had extension studies (total of 6 months use for pediatric and 18 months for adults) that showed maintenance of this improvement: +9 and +10 percentage points for adolescents/adults and children, respectively.^21,22^ Exposure to hypoglycemia remained low throughout our analysis period with median time below range (TBR) ∼1%; this compares favorably with the published RCTs with TBR equal to ∼1.6% for both. Although there was a statistically significant increase (due to the very large sample size) in time <54 mg/dL, from 0.1% to 0.15% median, this increase was irrelevant clinically (equivalent to <1 min/day).

The TIR improvement (and the maintenance of TBR) appeared within the first 2 weeks of use and were maintained throughout the 1-year analysis, showing consistency of glycemic control outside of any clinical trial structure. These results were achieved after consistently high use of closed loop: from 95% median time in closed loop for the first 2 weeks to 94% by year's end. The smaller T2DM sample (*n* = 378) also showed an improvement of 8 percentage points from 67% to 75% average TIR (median 70% vs. 78%) with very low hypoglycemia exposure: median TBR of 0.3%.

Other observational studies in real-world clinical use have reported glycemic outcomes and CLC use for commercial AP systems in T1DM (although not yet in T2DM). In 2019, Akturk et al. presented data from 127 patients of a single U.S. clinic transitioning from SAP therapy to AP and followed for 6 months.^23^ TIR improved ∼11 percentage points (from 59.5% to 70.1%) with TBR of 2.2% (although improved from 3.2% at baseline). Time in closed loop was ∼77% at 3 months, and 73% at 6 months with a significant decline in men only.

Berget et al. presented outcomes from a 6-month analysis of 92 children and young adults starting the same AP system at a single U.S. clinic: TIR improved almost 7 percentage points maintaining TBR at ∼3% (no change between baseline and AP use)^24^ whereas use of closed loop (a.k.a. auto mode) was only 65.5% at month 1 and 51.2% by month 6. A large proportion of the studied patients (*n* = 28, 30%) discontinued use of closed loop by 6 months, confirming the results presented in Lal et al.^25^ in a smaller (*n* = 68) but broader (9–61 years old) population.

It is important to note that the population studied in Berget et al. is particularly challenging and although the age range was represented in our analysis, we do not present outcomes by this specific subpopulation; nonetheless the school-aged children and adolescent subpopulation outcomes reported in this study compare favorably with Berget et al. In a similar analysis, Petrovski et al. presented data from 12 months of AP use in a single clinic in Qatar.^26^ In this pediatric observational study following a 30 participants prospective study of AP use in patients originally using multiple daily injections (MDI), TIR improved from 47% at baseline to 56.3% (Manual model) to 73% for 12 months of AP use, maintaining a high (compared with Berget et al.) closed-loop use of 86% at 12 months.

Limitations of our analysis include (1) the absence of HbA1c measurements, which limited our capacity to assess glycemic control to only CGM-based outcomes; (2) the relatively recent commercial approval of Control-IQ, biasing our sample toward early adopters, and (3) our requirement for a baseline CGM period before AP initiation. The latter, though it allowed for a more thorough characterization of our population, also likely biased our sample toward patients already comfortable with the use of technology in their diabetes management, with almost 99% prior Basal-IQ users. Finally, the nature of a retrospective observational study (especially the absence of a control group) does not allow to allocate the presented improvement solely to system use, but only to characterize glycemic control on the studied population after 12 months of use.

## Conclusions

In this article, we present analysis of data from the t:connect database (Tandem Diabetes Care) of Control-IQ technology users, who had available CGM data before initiation of Control-IQ technology and then had 12 consecutive months of real-life Control-IQ technology use. More than 9000 individuals met the preset CGM availability thresholds, generating nearly a billion data points that were included in this analysis. The results of this large-scale exploration of the real-world use of Control-IQ technology confirm the conclusions reached by the two pivotal trials of this system, in adolescents/adults and children, respectively: the use of Control-IQ technology increased time in the 70–180 mg/dL range by >10 percentage points from baseline.

This increase was evident after the first 2 weeks of use and was sustained throughout the entire year. In parallel, the frequency of CGM readings <70 mg/dL was kept low at ∼1% throughout the year. Time in closed loop was high throughout the year, with median use at 94%. We can, therefore, conclude that Control-IQ technology is well accepted and sustainably used by people over a broad age range, with varying degrees of baseline glycemic control, and different types of diabetes, achieving results that exceed the criteria for quality of glycemic control set forth by the International Consensus on TIR.^11^

## Supplementary Material

Supplemental data

Supplemental data

Supplemental data

Supplemental data
