# Supplementary material for: One Year Real-World Use of the Control-IQ Advanced Hybrid Closed-Loop Technology
Source: Diabetes Technol Ther. 2021 Sep 1;23(9):601–8. doi: 10.1089/dia.2021.0097 (PMC8501470; doi:10.1089/dia.2021.0097)
Supplement: Supplemental data [file Supp_Table1.docx]

**Table S1.** Comparison of glycemic outcomes for system users by age cohort (Baseline vs. 12-month use of Control-IQ technology (CIQ)). Data are expressed as median (IQR) unless otherwise specified.

|  | **6-13 years** | | **14-18 years** | | **19-63 years** | | **64 years and over** | |
| --- | --- | --- | --- | --- | --- | --- | --- | --- |
| No. of Participants | 716 | | 905 | | 5616 | | 1773 | |
| Diabetes duration (years) | 5 | | 7 | | 22 | | 34 | |
|  | Baseline | 12-mth Control-IQ | Baseline | 12-mth Control-IQ | Baseline | 12-mth Control-IQ | Baseline | 12-mth Control-IQ |
| Mean (SD) Sensor Glucose mg/dL | 181.5 (±30) | 166 (±21) | 183.6 (±34) | 167 (±24) | 166.1 (±31) | 153 (±20) | 158.7 (±24) | 147 (±16) |
| Sensor Time in Range | 53.5 (43.1- 64) | 64.7 (58.2- 72.1) | 53.3 (40.2- 66.1) | 65.4 (56.6- 73.4) | 64.5 (50.8-76.3) | 74.3 (65.2- 82.8) | 70.0 (57.9-80.8) | 79.0 (71.1- 85.5) |
| Sensor Time <54mg/dL | 0.1 (0-0.3) | 0.2 (0.1-0.4) | 0.1 (0-0.3) | 0.2 (0.1-0.3) | 0.1 (0-0.3) | 0.2 (0.1-0.4) | 0.1 (0-0.2) | 0.1 (0-0.3) |
| Sensor Time 54-70mg/dL | 0.9 (0.4-1.98) | 0.9 (0.5-1.6) | 0.8 (0.3-1.7) | 0.8 (0.4-1.5) | 0.9 (0.3- 0.02) | 0.01 (0- 0.02) | 0.01 (0- 0.01) | 0.01 (0- 0.01) |
| Sensor Time 180-250 mg/dL | 27 (22.1- 31.8) | 22.1 (19- 25.4) | 26.9 (21.8-31.7) | 22.3 (19- 25.5) | 24.8 (17.6- 31) | 19.4 (14-24.3) | 23.1 (14.8- 30.2) | 16.8 (11.7-22.1) |
| Sensor Time >250 mg/dL | 15.9 (8.8-23.9) | 10.5 (6- 15.6) | 15.4 (7.7-27.2) | 9.8 (5.2-16.4) | 7.6 (2.8- 0.15.5) | 4.3 (1.8-8.7) | 4.5 (1.5- 10.6) | 2.5 (1-5.4) |
| Time in Closed Loop Automation |  | 94.4(91.8-96.2) |  | 92.5 (88.3-95) |  | 96.4 (91.8-96.2) |  | 95.9 (93.3-97.4) |
