# Supplementary material for: One Year Real-World Use of the Control-IQ Advanced Hybrid Closed-Loop Technology
Source: Diabetes Technol Ther. 2021 Sep 1;23(9):601–8. doi: 10.1089/dia.2021.0097 (PMC8501470; doi:10.1089/dia.2021.0097)
Supplement: Supplemental data [file Supp_Fig3.docx]

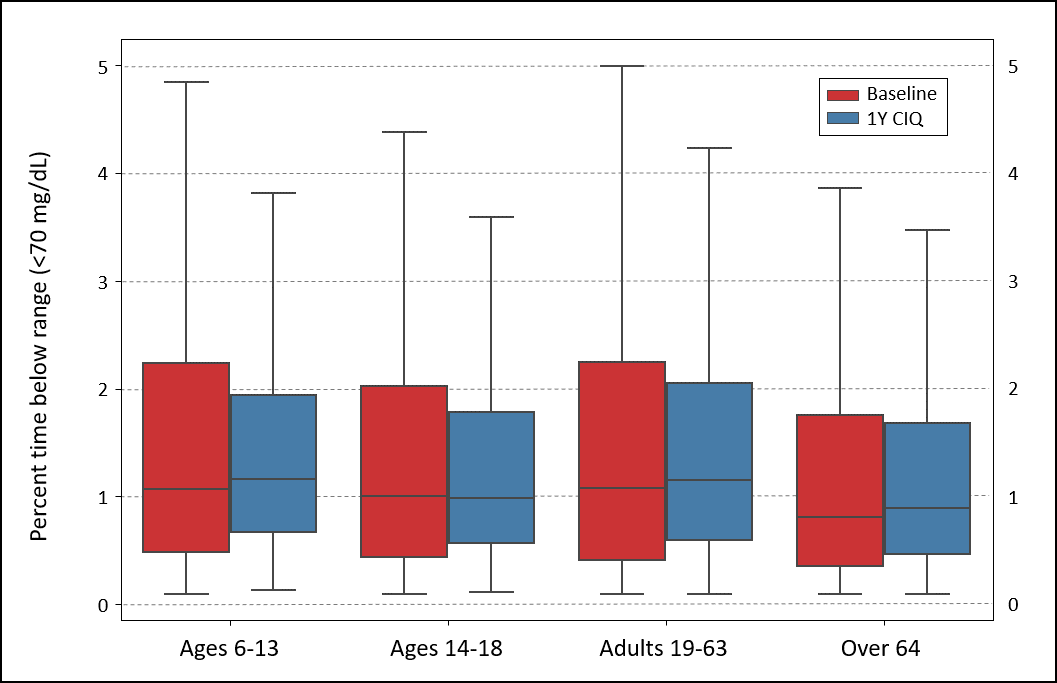


FigureS3. Changes in percent sensor TBR (below 70 mg/dl) from baseline to 365 days of Control-IQ technology use by age cohort.
